# Microfungal flora of *Apis mellifera anatoliaca* (Hymenoptera: Apidae) and *Varroa destructor* (Mesostigmata: Varroidae) from the Eastern Black Sea Region and fungal vector capacity in honey bee colonies

**DOI:** 10.1099/acmi.0.000626.v4

**Published:** 2024-02-16

**Authors:** Mehtap Usta

**Affiliations:** ^1^​ Trabzon University, Tonya Vocational School, Trabzon, Turkey

**Keywords:** *Apis mellifera anatoliaca*, fungal flora, honey bee health, microbiology, *Varroa destructor*

## Abstract

Honey bees have a great economic importance both in Turkey and in the world due to the products they produce and their contribution to pollination. For this reason, many microflora and microbiota studies have been conducted on bees. While these research were primarily focused on pathogen isolation, the ecological roles of non-pathogenic flora members and how they may be used are now being studied more extensively. Considering the importance of pathogens, the number of studies is expected to continue to increase. This study was carried out to determine the microfungal flora of the body surfaces and digestive tracts of dead honey bee (*Apis mellifera anatoliaca*) and *Varroa destructor* samples taken from different apiaries in the Eastern Black Sea Region of Turkey (Gümüşhane, Trabzon, Artvin and Ordu) in 2022. As a result of the study, a total of 11 different fungal species belonging to the genera *Penicillium*, *Alternaria*, *Mucor*, *Trichoderma*, *Fusarium*, *Aspergillus* and *Verticillium* were identified and the relationships of these fungi with bees were discussed based on the literature.

## Data Summary

No supporting external data were generated for this work.

## Introduction

Bees belong to the Apiformes group of the superfamily Apoidea in the order Hymenoptera [1]. There are around 25 000 recognized bee species worldwide [2]. Bumblebees are non-honey bee (*Apis mellifera*) species. However, in addition to *Apis mellifera*, there are around 10 000 other honey bee species worldwide, most of which are found in Far Eastern countries. Turkey’s climatic conditions, topography and location promote a richness of flora and other living species, making the bee fauna extremely diverse. Özbek [3] estimates that Turkey has around 2000 bee species.

Bees create and provide honey, beeswax, royal jelly, bee venom, and propolis to humans. Another benefit is that they pollinate cultivated plants that require allogamy (counter-pollination) alongside wild bees, ensuring a greater number and quality of crop yields [4–6].

*Apis mellifera* and the substances it produces are accompanied by a variety of microbes such as bacteria, moulds and yeast. More than 6000 micro-organisms have been isolated and identified from bees and their products. Fungi and yeasts were found to be more frequent than bacteria in bee isolates [7].

Fungal isolates from bees are critical for understanding and treating illnesses affecting bee populations. The fungus *Aspergillus fumigatus* has been a major focus of bee health study due to its link to nosemosis, or ‘nosema disease’, in Western honey bees (*Apis mellifera*) [8]. Isolation of fungal strains that are harmful to bees is critical for several investigations, including population genetics, illness development and ecological competition [9]. Additionally, the close relatedness of the black queen cell virus (BQCV) isolated from the fungus *Ascosphaera apis* to bee samples emphasizes the possibility for viral crossover between fungal and bee species [10]. *Ascosphaera apis*, which causes chalkbrood disease in honey bees, has been commonly observed in temperate countries, including Brazil [11]. *Melipona scutellaris* bees are vulnerable to the entomopathogenic fungus *Beauveria bassiana*, emphasizing the need to study how fungal infections affect diverse bee populations [12].

Understanding the function of fungi in bee health is critical to creating appropriate management measures. For example, *Beauveria bassiana*, an entomopathogenic fungus, has been studied with regard to treating *Varroa* mite infestations in honey bee colonies [13]. Furthermore, the possible ecological relevance of *Monascus* strains isolated from stingless bee colonies highlights the need to study the relationships between fungi and various bee species [14]. The discovery of honey bee genes that are differently expressed in response to infection with the chalkbrood fungus, *Ascosphaera apis*, has shed light on the molecular processes driving bee–fungus interactions [15]. Furthermore, the transit of fungi from the genus *Ascosphaera* to North America, together with bees from their original habitat in Japan, demonstrates the possible transcontinental movement of bee-associated fungi [16].

According to Doğan *et al*. quoted in Benjamin *et al*. [17], most fungi have various forms of symbiotic relationships with mites and other arthropods [18]. This association may be fairly clear at times. In other circumstances, a microscopic study of insects, thorough dissection, or observation of fungus-infected species throughout their life cycle is sufficient to detect the presence of a fungus. Fungi that can spread through these relationships are necrotrophic and biotrophic parasites [19]. Insects are often hosts to microbial symbionts. Although much symbiosis research has focused on bacterial communities, insect–fungus connections are also widespread. These relationships have important roles in insect nutrition and defence [20–22]. In other interactions, the insect uses the fungus directly as food or as a source of enzymes [23]. Some of these fungi are carried by arthropods in their environment [24–26]. The species of micro-organisms isolated from bees show great diversity. The number and diversity of micro-organisms isolated from worker bees collecting pollen and nectar is higher than from bees remaining in the hive. This is because bees harvesting pollen are subject to a diversity of micro-organisms by their activity outside the hive than bees within the hive. Larvae ingest these micro-organisms by eating contaminated food. Bees transmit this flora to each other during pollen consumption and food exchange with other bees in the colony [7]. Fungal spores can be found in bee pollen, combs, honey and on all surfaces within the hive. Spores can survive for years and can be a continuous source of infection [27–29]. Bee-associated fungi, particularly pathogenic fungi [30], are described as those that are saprophytic on stored bee resources or products or that harm bee health. *Aspergillus* P. Micheli ex Link, *Penicillium* Link and *Mucor* Fresen species are known to be secondary invaders of bee larvae and prepupae [31, 32]. Fungi belonging to this genus (*Aspergillus*) are usually found in the digestive tract of worker bees. *Penicillium glabrum* (Wehmer) Westling, *Penicillium aurantiogriseum* Dierckx, *Aspergillus flavus* Link and *Aspergillus niger* var. *niger* Tiegh are usually the isolated species belonging to these genera [33, 34]. Other fungi isolated from the intestines of worker bees include *Cladosporium cladosporioides* (Fresen) G.A. de Vries and *Alternaria tenuissima* (Kunze) Wiltshire [7]. In addition, fungi belonging to the genera *Penicillium*, *Aspergillus*, *Mucor* and *Trichoderma* Persian have been isolated from many surfaces such as dead bees in the colony, combs, bee food, honey and pollen [35]. Frequent isolation of fungi from beehives is closely related to the temperature and humidity conditions inside the hive [36].

Benoit *et al*. investigated the microfungal flora of *Varroa destructor*, a mite harmful to honey bees, and found that this flora consists of cosmopolitan soil saprophytes such as *Aspergillus*, *Penicillium*, *Fusarium* Link, *Trichoderma*, *Alternaria* Nees, *Rhizopus* Ehrenb and *Mucor* [37]. According to Muz, *Varroa destructor* mites feed on the haemolymph of the bee [38]. Meanwhile, the wounds opened by the mite provide ease of passage for microbial pathogens vectored in the bee body.

Özakın *et al*. also examined old and new combs bacteriologically and mycologically and isolated *Aspergillus fumigatus* Fresen, *Cladosporium corrioni* and *Penicillum* spp. as fungal flora [39]. Özkırım and Keskin isolated *Ascosphaera apis* (Maasen ex Claussen), the agent of lime disease, and *Aspergillus flavus*, the agent of stone disease, from honeycomb samples taken from Ankara province and its surroundings [40].

It has been reported that some fungi belonging to genera such as *Fusarium*, *Penicillium*, *Rhizopus* and *Aspergillus* occasionally spoil nutrients in the hive and cause the extinction of natural populations due to decreases in the food source [35].

In Turkey, fungal isolation studies have been carried out on bee products, but there are insufficient mycoflora studies on bees. In this study, the microfungal flora of the body surface and digestive systems of honey bees (*Apis mellifera*) taken from different beekeepers in the Eastern Black Sea Region was determined with regard to bee–fungus relationships. Various aspects of entomopathogenic, phytopathogenic, parasitic and antagonistic properties are discussed based on the literature. Thus, this paper contributes to the limited number of microflora studies on bees and their isolated fungal species. In addition, fungal vector potential against pests was evaluated by isolating fungi from the surface of *Varroa* mites.

Apart from pathogenic interactions, the observation of fungi in bees is generally recognized as a sign of stress. Bees stressed by nosemosis, xenobiotics or high overwintering temperatures harbour more fungi in their gastrointestinal tract [41–47]. However, it is increasingly recognized that not all fungi associated with bees are indicative of disease or decay, and some may be commensal or even mutualistic [48, 49]

Bee fungal isolations are critical to understanding the dynamics of bee–fungus interactions, disease transmission and the formulation of control plans. The wide range of fungi that impact various bee species highlights the importance of conducting extensive studies to protect bee populations and their critical function in ecosystems. This study aimed to determine whether the fungi obtained from *Apis mellifera anatoliaca*, the most abundant species in Turkey, and *Varroa destructor* mites, which causes the greatest damage to honey bees, come from natural flora or herbal factors, and we aimed to develop natural control methods for the disease-causing fungi. The potential of the fungi obtained, especially on mites, will be tested in future studies.

## Methods

### Collection of honey bees and varroa mites and isolation of fungi

Dead bees collected from the hive front board were brought to the laboratory under sterile conditions. Live honey bees collected were not used in the experiments. The reason for the use of dead honey bees was that fungal death was suspected. Honey bee and *Varroa* mite samples were collected from the provinces of Gümüşhane, Trabzon, Artvin, and Ordu based on observations of people with an interest in beekeeping. For the outer surface flora, the bees were mixed by placing them in glass tubes containing 5 ml of sterile physiological water (Adeka), and each of these mixtures was placed in Petri dishes containing potato dextrose agar (PDA) [15]. Physiological water used in the study is a commercial product used for medical purposes. To determine the intestinal flora, surface sterilization of the bees taken from the glass tubes was ensured, and intestines removed by dissection under aseptic conditions were homogenized and left on the PDA medium [50]. Petri dishes containing PDA were incubated at 25 °C under aerobic conditions for 1 week and colonies with different colours or morphologically different appearances were isolated. The isolates with different colony morphology were purified. PDAY (potato dextrose agar +1 % yeast extract) was used as the first medium during purification. Ampicillin (50 µg ml^−1^), streptomycin (200 µg ml^−1^) and tetracycline (20 µg ml^−1^) were added to the medium to avoid bacterial growth [51]. Glycerol stocks of pure fungal samples were prepared and stored at −80 °C for used in the studies.

*Varroa* mites were collected from hives and were brought to the laboratory under sterile conditions. No fungi were seen on the mites, either macroscopically or microscopically (100×), and all the mites appeared healthy. The mites (in three batches) were rinsed twice for 1 min in 1 ml fresh deionized (DI) water in cap bottles and transferred to a 9 cm Petri dish with PDA. Plates were incubated for 7 days at 22–24 °C with daily observations for fungal growth. Hyphae tips, visible under a dissecting microscope, were excised using a sterile scalpel and subcultured in PDA. Petri dishes containing PDA were incubated at 25 °C under aerobic conditions for 1 week and those isolates with different colours or morphologically different appearances were isolated. The isolates with different colony morphology were purified. PDAY was used as the initial medium during purification. Ampicillin (50 µg ml^−1^), streptomycin (200 µg ml^−1^) and tetracycline (20 µg ml^−1^) were added to the medium to avoid bacterial growth [51]. Glycerol stocks of pure fungal samples were prepared and stored at −80 °C for use in the studies.

### Molecular identification

DNA was extracted from the mycelium after 14 days of growth on PDAY medium at 28 °C. DNA isolation was performed using the QuickDNA Fungal/Bacterial MiniPrep Kit (Zymo Research). Isolation was carried out in line with the kit’s instruction booklet. The partial sequence of the ITS1–5.8S–ITS2 gene region was amplified by PCR using primers ITS5 (5′-GGAAGTAAAATCGTAACAAGG-3′) and ITS4 (5′-TCCTCCGCTTATTGATATGC-3′) [52]. The ITS1–5.8S–ITS2 gene region is a widely used region for species identification [53]. PCR mixes contained 50 ng DNA template, 10 µl 5× Phusion reaction buffer, 200 µmol dNTPs, 1 µl (50 pmol) of each primer, and 1 unit of PhusionDNA polymerase in a total volume of 50 µl. PCR conditions were set as described in the PCR kit using a thermal cycler (Bio-Rad).

**Fig. 1.**
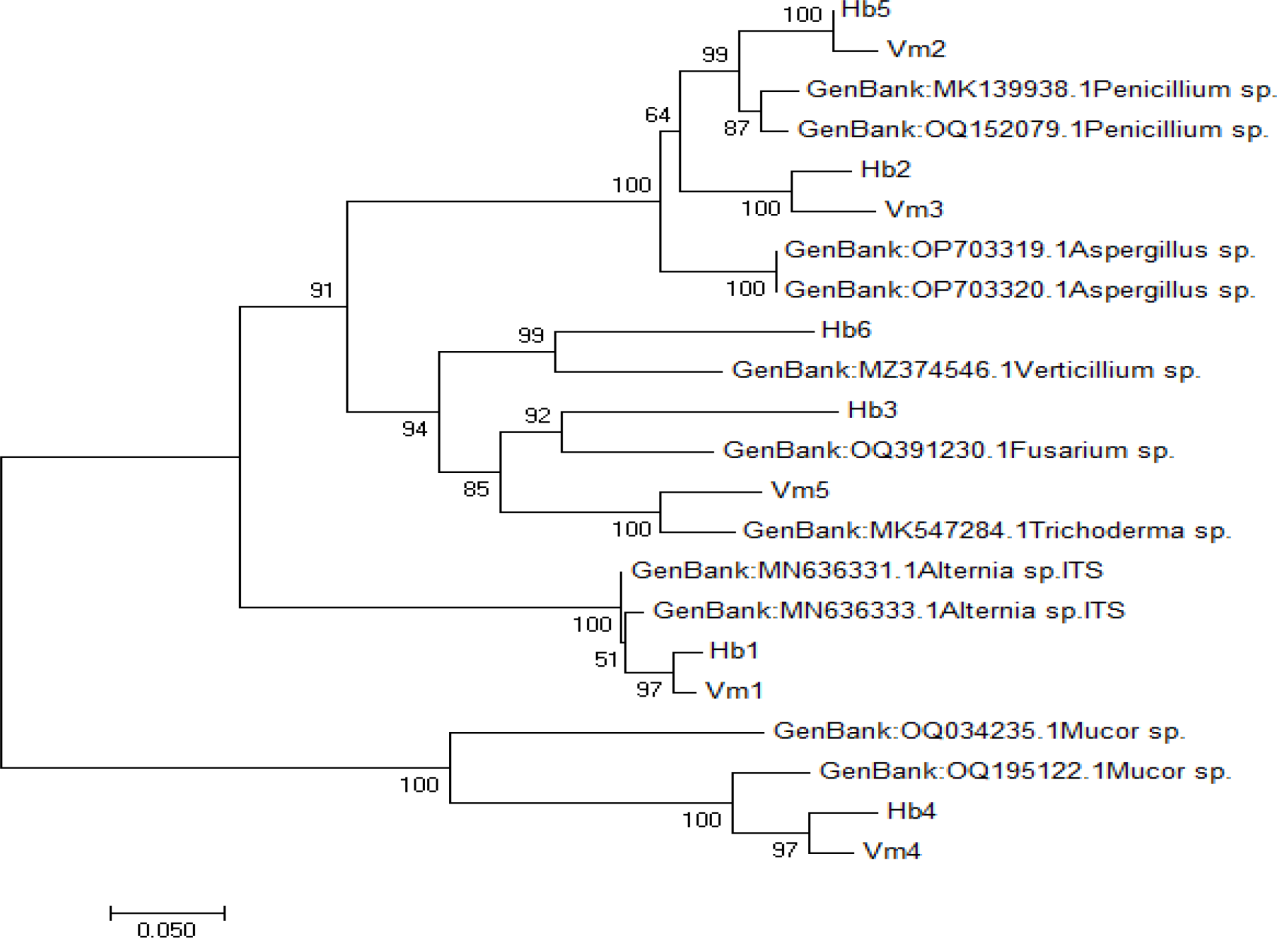
Evolutionary relationships of the taxa studied. The evolutionary history was inferred using the neighbour-joining method [99, 100]. The optimal tree with the sum of branch length=2.05567204 is shown. The percentage of replicate trees in which the associated taxa clustered together was determined in a bootstrap test (1000 replicates) [101].

PCR products were sent to Sentebiogen (Ankara) for DNA sequence analysis. To validate species identification, the resultant DNA sequences were matched with DNA sequences in the NCBI GenBank using the Blast tool, and then utilized for phylogenetic analysis [54]. The program mega 7 [53] was used for phylogenetic analysis (Fig. 1). Table 1 lists the GenBank accession numbers for each sequence.

**Table 1.** Percentage query coverage, level of similarity and accession numbers of the isolated fungi based on blast searches in NCBI GenBank using ITS gene sequences

Fungus host	Isolate name	Fungus name	Accession no.	Query coverage (%)	Similarity (%)
Honey bee	Hb1	*Alternaria* sp.	OQ692417	100	96.05
Honey bee	Hb2	*Aspergillus* sp.	OQ692419	99	98.0
Honey bee	Hb3	*Fusarium* sp.	OQ692421	98	96.85
Honey bee	Hb4	*Mucor* sp.	OQ692422	99	98.45
Honey bee	Hb5	*Penicillium* sp.	OQ692424	99	99.80
Honey bee	Hb6	*Verticillium* sp.	OQ692427	98	98.66
Varroa mite	Vm1	*Alternaria* sp.	OQ692418	99	97.88
Varroa mite	Vm2	*Penicillium* sp.	OQ692425	99	98.41
Varroa mite	Vm3	*Aspergillus* sp.	OQ692420	100	98.01
Varroa mite	Vm4	*Mucor* sp.	OQ692423	98	98.93
Varroa mite	Vm5	*Trichoderma* sp.	OQ692426	98	98.24

## Results and discussion

As a result of the isolation procedure, 150 colonies were obtained from the surfaces and intestines of honey bees; 45 of them were determined as yeast and were not included in the study. As a result, six different genus-level fungi were determined and registered to NCBI GenBank; registration numbers are given in (Table 1). Phylogenetic analysis was performed by using mega 7 (Fig. 1). The results of phylogenetic tree analysis also support the molecular analysis, phylogenetic analysis showing high similarity rates to the same genera. It is expected that samples isolated from both *Varroa* mites and honey bees and belonging to the same genus will be similar under phylogenetic analysis. Fungi were determined at five different genus levels from *Varroa* mites. They were also registered with NCBI GenBank; registration numbers are shown in (Table 1). *Alternaria* sp., *Trichoderma* sp. and *Penicillium* sp. were isolated from the surfaces and intestines of dead bees. *Verticillium* sp. from the surfaces of dead bees, *Mucor* sp., *Aspergillus* sp. and *Aspergillus* sp. from the intestines of dead bees, the surface of dead bees and the gut of dead bees and *Fusarium* sp. and *Mucor* sp. from the surfaces of dead bees.

The isolation of *Alternaria* sp., *Trichoderma* sp., *Penicillium* sp., *Verticillium* sp., *Mucor* sp., *Aspergillus* sp. and *Fusarium* sp. fungi from bees has important consequences for bee health and ecological relationships. The wide variety of fungi recovered from bees demonstrates the complexities of bee–fungus interactions and their potential influence on bee populations. For example, various studies have reported the isolation of *Penicillium* sp. and *Aspergillus* sp. from bees, highlighting the prevalence of these fungi in bee-related environments [37, 55–59]. Additionally, the presence of *Mucor* sp. and *Trichoderma* sp. in bee samples highlights the need to study their ecological significance in bee habitats [60–64].

Furthermore, the isolation of *Fusarium* sp. and *Alternaria* sp. from bees raises concerns regarding their possible pathogenicity and influence on bee health. These fungi have been linked to plant illnesses and may influence bees via in variety of ways, including direct pathogenicity and secondary metabolite synthesis [65, 66]. Furthermore, the presence of *Verticillium* sp. in bee settings emphasizes the need to examine the possible connections between this fungus and bee populations, given its known pathogenicity in plants [59].

The isolation of these fungi from bees also raises questions about the sources of exposure and transmission routes. Understanding how bees come into contact with these fungi and the potential implications for bee health is crucial for developing effective management strategies. Moreover, the potential for these fungi to interact with other micro-organisms in the bee microbiome underlines the complexity of bee–fungus interactions and their ecological significance [55, 67, 68].

Low-molecular-weight compounds formed as a result of secondary metabolism of fungal genera such as *Aspergillus*, *Penicillium*, *Fusarium*, *Alternaria* and *Claviceps* are termed mycotoxins. These compounds have various toxic effects on human and animal health. Fungi producing mycotoxins can be found ubiquitously (such as various layers of the atmosphere) by being transported by wind and air. The presence of mycotoxins may vary according to climatic conditions, type of product, geographical location, season and year [69].

*Aspergillus* and *Penicillium* are common in honey bee colonies [7]. In the present study, specimens belonging to these genera were isolated. *Aspergillus* sp. and *Penicillium* sp. were isolated from the surfaces and digestive systems of dead bees. In addition, *Aspergillus* sp. was isolated ifrom *Varroa* mites, which may indicate the possibility of transmission through adult honey bees. However, *Aspergillus* sp. is most likely to access new brood cells via conidia carried on mites that enter the hive in search of a new host bee. Thus, in addition to causing heavy losses in the honey bee industry through direct invasion [70], *Varroa destructor* may also promote the spread of stone brood disease, as first suggested by Liu [71]. In previous studies, *Aspergillus niger* was isolated from the digestive systems of worker bees, and *Aspergillus fumigatus* from honeycombs; *Aspergillus flavus* was isolated from both the digestive systems of worker bees and honeycombs [7, 39, 40]. *Penicillium* species, which were found in four separate isolation studies, have been isolated from many surfaces such as dead bees, hives, pollen, honey and honeycombs [35]. Accordingly, the species found in honeycombs and beehives in previous pstudies were expected in the surface flora of bees in this study, and the species found in honey and pollen were encountered in the digestive systems of bees. In addition to these studies, fungi in the genus *Aspergillus* can be opportunistic pathogens of both adult bees and larvae [31] and produce mycotoxins that can be toxic to bees [34, 33]. Bees are also associated with moulds, fungi including the genera *Aspergillus*, *Penicillium* and *Cladosporium*, which grow by spread of mycelia. Unlike yeasts, moulds are generally not very selective in their habitat. They can grow by utilizing various carbon sources of energy. Moulds are often found in flower pollen and stored pollen matter. Within these bee-associated genera there are many different varieties of fungi. They inhabit a wide variety of habitats and have different ecological roles, including plant pathogens (e.g. *Alternari*a), bee pathogens (e.g. *Aspergillus flavus* [72]; *Ascosphaera* [30]).

*Mucor* and *Fusarium* species were isolated from the surface flora of dead bees. Spores of these fungi were not found in their digestive systems. *Mucor* species have been isolated from many surfaces, dead bees, pollen, and bee larvae in hives [35]. Glifiski and Buczek defined *Mucor* species as secondary invaders of bees [32]. This genus is known to include soil-borne cosmopolitan and saprophytic species [73]. Therefore, *Mucor* species are likely to be found in the surface flora of bees and show saprophytic development after the death of bees. Fungi may also benefit bees by reducing the growth of pathogens in bee products or micro-organisms that cause spoilage in bee products through competition between microorganisms, but the literature on this is complex and is not clear [74]. For example, *Mucor*, *Penicillium*, *Aspergillus*, *Talaromyces*, *Rhizopus* and *Cladosporium* isolated from honey bee products and guts inhibited the growth of *Ascosphaera apis* [75, 76].

*Fusarium* species are known to be isolated from hives [35]. *Fusarium* species cause fungal diseases known as *Fusarium* wilt in agricultural products [77]. Accordingly, it is thought that the bees may have taken fungal spores from the plants they visited to collect pollen and nectar and carried them to the hive. Fungi of the genera *Fusarium*, *Cladosporium* and *Alternaria* are known to be predominant in the mycobiota of forage legumes. As long as bees visit these plants, they will transfer contaminated pollen grains to the hives [78, 79].

*Trichoderma* and *Alternaria* species have been isolated from *Varroa* destructor mites, which is a parasite of honey bees [37]. In addition, *Trichoderma* species are common in bee colonies, in addition to *Aspergillus*, *Penicillium* and *Mucor* species [35].

Some fungi are known to have antagonistic properties to plant pest fungi. *Trichoderma* species are preferred in biological control because they accelerate plant development, make plants resistant to soil-borne pathogens by stimulating plant defence mechanisms, and produce various antibiotic compounds, and many commercially produced preparations exist [80, 81, 82, 83]

According to Kapongo *et al*. honey bees and bumblebees have been used effectively as vectors of fungi that suppress plant pathogens [84]. In this study, *Trichoderma* species were isolated from *Varroa* mites, and *Trichoderma harzianum* is known to be an antagonistic fungus used in biological control against *Botrytis cinerea* Pers. By using it as a vector, *Trichoderma harzianum* was delivered to the target plant and a significant yield increase was achieved [85–87]. The use of *Trichoderma* species as biocontrol agents has been investigated for over 70 years, but the commercial use of strains has gained importance in recent years. As biocontrol agents, *Trichoderma* spp. are particularly effective against fungi of the *Ascomycetes*, *Deuteromycetes* and *Basidiomycetes* groups, which contain soil-borne and even air-borne pathogens. Many strains such as *T. harzianum*, *T. viride*, *T. virens* (*Gliocladium virens*), *T. aureoviride*, *T. pseudokoningii*, *T. koningii* and *T. longibrachiatum* have been identified for use as potential biocontrol agents and the fungi they affect have been determined [88, 89].

The phytopathogenic fungus *Alternaria alternata*, which is abundant in soil and rotten plant material, causes ‘alternaria brown rot’ in many plants [90]. Some *Alternaria* species are causal agents of many plant diseases commonly known as black spot, brown spot, *Alternaria* spot, leaf spot and stem canker in cereals, oilseeds, fruits and vegetables. These diseases caused by pathogenic species and the toxins they produce greatly affect structures that have important roles in cell metabolism such as mitochondria, chloroplasts, plasma membranes, Golgi complex and nucleus [91, 92] In this study, *Alternaria* sp. was isolated. This suggests that honey bees are carriers of *Alternaria* sp. Isolates of the genus *Alternaria* isolated from nature, agricultural products or manufactured foods and developed under laboratory conditions have been found to be toxic in studies on rats [93], chicken embryos [94] and human cells [69, 95].

*Verticillium* is a genus that harbours a variety of plant and insect pathogens. *Verticillium* sp. was isolated from the surface of dead bees. Among the mycoflora studies on bees, no isolation of *Verticillium* species was previously reported. Isolation of *Verticillium* isolation was done in a study conducted in 2010 [96]. However, the presence of plant pathogenic and insect pathogen species in this genus suggests that such fungi will be encountered in isolations from bees. At least 700 species of entomopathogenic fungi belonging to at least 90 genera have been described. Some species such as *Verticillium lecani* (Zimm.) (candlestick mould), *Beauveria bassiana* (Bals.) Vull. (insect mould), *Isaria fumosorosea* (Wize) Brown [pleasant isarya (=*Paecilomyces fumosoroseus*] and *Metarhizium anisopliae* (Metch) Sorok (insectivore) are commercially produced and used for the control of different pests in many countries [97, 98].

## Conclusion

Numerous studies have aimed at the discovery of micro-organisms found associated with bees and investigated the tasks of these micro-organisms. However, these studies aimed to increase bee products by eliminating pathogens and parasites, and also to distribute those that are not pathogenic to bees and have the potential to be used in the biological control of pests through bees.

In this study, one of the isolated fungi is *Aspergillus* sp., known for causing stone disease in bees. Except for this species, no fungi harmful to bees were identified. Consequently, our evaluation of the bee–fungus relationship extends beyond pathogenicity considerations. There is a pressing need for further research to uncover additional micro-organisms that do not harm bees but may have remained undetected. Our contribution aims to enrich the understanding of the mycoflora specific to bees in the Eastern Black Sea Region, a crucial honey source area in Turkey. Subsequent research endeavours will focus on exploring the potential of *Varroa* as a fungal vector and isolating additional fungal strains important for both *Varroa destructor* and *Apis mellifera*.

In conclusion, the isolation of *Alternaria* sp., *Trichoderma* sp., *Penicillium* sp., *Verticillium* sp., *Mucor* sp., *Aspergillus* sp. and *Fusarium* sp. fungi from bees emphasizes the need for extensive investigation to understand their roles in bee health, disease dynamics and ecological interactions. Understanding the effects of these fungi on bee populations is critical for establishing measures to protect bee health and maintain their key function in ecosystems.
